# The Role of Mitochondrial Non-Enzymatic Protein Acylation in Ageing

**DOI:** 10.1371/journal.pone.0168752

**Published:** 2016-12-29

**Authors:** Shin Yee Hong, Li Theng Ng, Li Fang Ng, Takao Inoue, Nicholas S. Tolwinski, Thilo Hagen, Jan Gruber

**Affiliations:** 1 Department of Biochemistry, Yong Loo Lin School of Medicine, National University of Singapore, Singapore, Singapore; 2 Department of Pharmacology, Yong Loo Lin School of Medicine, National University of Singapore, Singapore, Singapore; 3 Department of Science, Yale- NUS College, Singapore, Singapore; 4 Department of Biological Sciences, National University of Singapore, Singapore; Ludwig-Maximilians-Universitaet Muenchen, GERMANY

## Abstract

In recent years, various large-scale proteomic studies have demonstrated that mitochondrial proteins are highly acylated, most commonly by addition of acetyl and succinyl groups. These acyl modifications may be enzyme catalysed but can also be driven non-enzymatically. The latter mechanism is promoted in mitochondria due to the nature of the mitochondrial microenvironment, which is alkaline and contains high concentrations of acyl-CoA species. Protein acylation may modify enzyme activity, typically inhibiting it. We posited that organismal ageing might be accompanied by an accumulation of acylated proteins, especially in mitochondria, and that this might compromise mitochondrial function and contribute to ageing. In this study, we used *R*. *norvegicus*, *C*. *elegans* and *D*. *melanogaster* to compare the acylation status of mitochondrial proteins between young and old animals. We observed a specific age-dependent increase in protein succinylation in worms and flies but not in rat. Rats have two substrate-specific mitochondrial deacylases, SIRT3 and SIRT5 while both flies and worms lack these enzymes. We propose that accumulation of mitochondrial protein acylation contributes to age-dependent mitochondrial functional decline and that SIRT3 and SIRT5 enzymes may promote longevity through regulation of mitochondrial protein acylation during ageing.

## Introduction

Mitochondrial dysfunction and hypo-metabolism are well-documented features of the ageing process in many organisms, ranging from nematodes to humans [1–4]. Impaired mitochondrial function is also commonly observed with ageing in various model organisms [5–7]. Mitochondrial dysfunction, moreover, plays an important role in the development and progression of many age related diseases [8–11]. Detrimental mutations in genes involved in mitochondrial function and particularly in homeostasis reduce lifespan or hasten the onset of neurodegenerative diseases in a variety of animals, including humans [8,9,12–14].

The most widely proposed mechanism for ageing-associated mitochondrial dysfunction is oxidative stress [15–18]. Mitochondrial oxidative stress is mainly due to reactive oxygen species (ROS) generated by the electron transport chain (ETC). ROS can react with mitochondrial DNA and proteins, leading to the formation of adducts that, in turn, may impair the function of the ETC. It has been proposed that, as a result of ETC dysfunction, more ROS are produced and thus a vicious cycle of increased oxidative damage may ensue [19], however the evidence for this cycle is at best equivocal [20,21]. The causal role of ROS mediated mitochondrial damage in ageing has also been questioned as attempts to lower ROS levels (e.g. with antioxidants or by overexpression of ROS detoxifying enzymes) do not consistently extend lifespan, and because ageing is not always correlated with a consistent increase in markers of oxidative damage [22–27]. Therefore, although it is clear that mitochondrial function declines with age in most animals, the molecular cause for this decline remains controversial.

Recently, protein acylation has been identified as a mechanisms that is distinct from other forms of posttranslational protein modification but can also impair protein function [28–31]. Protein acylation is the addition of an acyl group, typically to the ε-amino group of lysine residues in proteins. The substrates for protein acylation are usually energy rich acyl-coenzyme A thioesters such as acetyl-CoA, succinyl-CoA and malonyl CoA, resulting in protein acetylation, succinylation and malonylation, respectively [32]. Protein acylation can be mediated enzymatically through the action of acetyl transferases and can be reversed through deacylating enzymes (HDACs) of the histone deacetylase family (comprising of various classes) and the Sirtuin family [33–35]. The best characterized functions of protein acetylation are chromatin regulation as well as transcriptional regulation through the acetylation of histone proteins and transcription factors [36,37]. In addition, it has been recognised that different forms of acylation of other cellular proteins can also regulate cellular processes, including autophagy and cellular metabolism [38–40]. Notably, the regulation of protein acylation, specifically through Sirtuin deacylating enzymes, has been recognized to play an important role in ageing at least in various model organisms [41,42].

Interestingly, recent proteomic studies have demonstrated that mitochondrial proteins, including many metabolic enzymes and ETC subunits, are highly acylated *in vivo* [29–31,38,43,44]. In functional studies, acylation of mitochondrial proteins was shown to most commonly inhibit protein or enzyme function but the exact mechanism and, in particular, the acyl transferases involved in mediating the acylation of mitochondrial proteins are currently unclear [28,45]. As originally described by Paik (1970), proteins can also be acetylated non-enzymatically[46]. Recent work has shown that mitochondrial protein acetylation and succinylation is likely predominantly mediated in a non-enzymatic manner [47–49]. Such non-enzymatic protein acylation in mitochondria is favoured by the high concentrations of acetyl-CoA and succinyl-CoA present in mitochondria and by the alkaline microenvironment in the mitochondrial matrix [32,47,48,50]. Of particular interest in the context of ageing is the notion that this non-enzymatic mitochondrial protein acylation may contribute to mitochondrial dysfunction during ageing and to the pathogenesis of various human diseases.

In this study, we set out to test some of the expected consequences of the hypothesis that mitochondrial dysfunction during ageing is associated with increased mitochondrial protein acylation levels. In order to quantify mitochondrial protein acylation, we have used a Western blotting based approach. First, we optimized and validated antibodies and extraction protocols for the quantification of protein acylation (acetylation and succinylation) in tissue extracts. After extensive antibody validation, we confirmed that conditions prevalent in mitochondria promote protein acylation and that this process can be reversed by recombinant SIRT3 protein. We then compared the mitochondrial acylation status in ageing nematodes (*Caenorhabditis elegans*), flies (*Drosophila melanogaster*) and rodents (*Rattus norvegicus*). In rats we compared acylation levels in brain, liver and heart tissue at different ages while in *C*. *elegans* we determined acylation levels at different ages in wild-type and long lived (*age-1*) mutants and compared ageing trends in acylation with protein carbonylation (oxidative damage). For ageing nematodes and rat brain tissue we also determined parameters of mitochondrial function (oxygen consumption) in tissues from ageing animals to quantify age-dependent decline in respiratory capacity. Our study shows that there is a specific increase in protein succinylation in both *C*. *elegans* and *D*. *melanogaster* but not in rats, at least for the ages tested. This change in acylation is associated with by a clear age-dependent decline in the metabolic activity of nematodes but not rat brains. Interestingly, protein succinylation in nematodes appears to show ageing trends that are at least as robust as those for protein oxidation. These results are consistent with the hypothesis that substrate-specific mitochondrial deacylases in rat ameliorate age-dependent increases in mitochondrial protein acylation and warrant further study in ageing models and humans.

## Material and Methods

### 2.1 *C*. *elegans*

The temperature sensitive sterile *Caenorhabditis elegans* strains TJ1060 [*spe-9(hc88) I; fer-15(b26) II*] and TJ1062 [*spe-9(hc88) I; fer-15(b26) age-1(hx542) II*] provided by *Caenorhabditis* Genetics Center (Minneapolis, MN), were used in this study. The nematodes were propagated at 15°C and maintained at 25°C on nematode growth medium (NGM) plates supplemented with *Escherichia coli* OP50-1 as food source [51]. All strains are infertile at 25°C. Age-synchronized animals were prepared by hypochlorite treatment. Eggs were allowed to hatch and age-synchronized animals were harvested at day 5 and day 12 of adulthood.

### 2.2 *D*. *melanogaster*

Fruit flies (*Drosophila melanogaster*, OregonR) of mixed gender were cultured on medium containing glucose (6g Bacto agar, 114g glucose, 56g cornmeal, 25g live yeast and 20ml of 10% Nipagin in 1l final volume). Ten age-synchronized 1-, 10- and 30-day-old flies were collected in micro-centrifuge tubes. Whole fly lysates were prepared by homogenizing ten flies in lysis buffer containing 280mM sucrose, 0.1% Triton-X, 10mM Tris-HCl (pH 7.4), 1mM EDTA, 0.5mM protease inhibitor PMSF, deacetylase inhibitors 500nM trichostatin A (TSA) and 10mM nicotinamide (NAM). SDS sample loading buffer was then added to the samples and subjected to SDS-PAGE and western blotting.

### 2.3 *R*. *norvegicus*

All animals tissues used in this study were surplus tissues from male rats (250– 350g) taken from the control groups of two experiments (one using Wistar and the other Sprague-Dawely rats) carried out at DSO Singapore National Laboratories. Access to food and drinks of all control animals was ad libitum and animals were kept on a 12h/ 12h light/ dark cycle. Animals were sacrificed by first anaesthetizing them with ketamine (75mg/kg) and xylazine (10mg/kg) intraperitoneally, followed by transcardial puncture. All animal experiments were approved by the DSO Institutional Animal Care and Use Committee (DSO IACUC).

### 2.4 Selection of age-groups

Nematodes of this strain and at the temperature used typically survive to a maximum age of 18–20 days with a median lifespan of about 10 days [52] while the average lifespan of fruit fly at 25°C is about 60 to 80 days, with maximum lifespan of 70 to 90 days [25,53]. For all animals we have chosen ages at which we expected to see significant age-dependent decline and damage accumulation but well before there is significant mortality and associated selection effects.

### 2.5 Immunoblotting

Proteins were resolved by SDS-PAGE and transferred onto nitrocellulose membrane (Bio-Rad Laboratories, Inc.). Antibodies used to probe the membrane were: mouse anti-GST, goat anti-pan acetyl antibody (sc-8649; Santa Cruz Biotechnology), mouse anti-acetyl lysine (05–515 clone 4G12; Millipore), anti-rabbit acetylated-lysine (#9441, CST), anti-rabbit succinylated-lysine (PTM Biolabs), mouse anti-NDUFS3 antibody (Abcam).

#### 2.5.1 Protein carbonyl content (PCC) determination

Protein carbonyl content was performed as described in [54,55]. Briefly, two hundred nematodes were assayed for protein carbonyl content. The nematodes were collected, washed in M9 buffer and sonicated on ice in PBST (phosphate-buffered saline with 0.1% Tween-20) containing 1mM PMSF. Protein concentration of the lysates was determined using Bradford protein assay (Bio-Rad Laboratories, Hercules, CA, USA). PCC of the lysates were determined using the Oxyblot Protein Oxidation Detection Kit (Milipore, Billerica, MA, USA). 2μg of lysate was derivatized and transferred onto a nitrocellulose membrane via slot blot (Bio- Rad, Hercules, USA) under vacuum. The membrane was blocked and probed with anti-dinitrophenylhydrazine primary antibody (1:150), followed by secondary detection with an anti-rabbit horseradish peroxidase- conjugated IgG antibody (1:300). Protein bands were visualized using a chemiluminescence substrate mixture (Pierce Biotechnology, Thermo Fisher Scientific, Waltham, MA, USA).

### 2.6 Preparation of recombinant Sirt3

GST-Sirt3 fusion protein plasmid was generated by inserting Sirt3 coding sequence (Accession: NM_012239) corresponding to 15–399 amino acids into the pGEX-KG vector. The fusion protein was then expressed in BL21 bacteria and purified using GSH-agarose beads (GE Healthcare).

### 2.7 Mitochondria extraction

#### 2.7.1 Mouse/Rat

Mitochondria were extracted from mouse or rat tissues by differential centrifugation as previously described [56] using mitochondrial isolation buffer that contained 280mM sucrose, 10mM Tris-HCl (pH 7.4), 1mM EDTA as well as the deacetylase inhibitors 500nM trichostatin A (TSA) and 10mM nicotinamide (NAM).

#### 2.7.2 *C*. *elegans*

Mitochondria from 10,000 nematodes were extracted as previously described [54]. Nematodes were washed in S-basal buffer (100mM NaCl, 5.7mM K_2_HPO_4_, 44.1mM KH_2_PO_4_, 0.01mM cholesterol) and homogenized in isolation buffer (210mM mannitol, 70mM sucrose, 0.1mM EDTA, 5mM Tris-HCl, pH 7.4). Debris and nuclei were removed from the homogenate by differential centrifugation at 600g for 10 min at 4°C. Mitochondrial pellet was obtained by centrifuging the supernatant at 7200g for 10 min at 4°C and re-suspended in Tris-EDTA buffer (50mM Tris-HCl, 0.1mM EDTA, pH 7.4). Mitochondria were kept at -80°C until further analysis.

### 2.8 Measurement of oxygen consumption

#### 2.8.1 Brain Extract

Oxygen utilization was measured using a Clark type oxygen electrode (Rank Brother Ltd). Mitochondria were resuspended in respiratory buffer consisting of 225mM sucrose, 10mM KCl, 1mM EDTA, 10mM K_2_HPO_4_-KH_2_PO_4_, 5mM MgCl_2_ and 10mM Tris- HCl (pH 7.4). Glutamate-malate, ADP and FCCP were added sequentially to measure the different respiratory states.

#### 2.8.2 *C*. *elegans*

Oxygen consumption rates of *C*. *elegans* were measured using Seahorse XF-96 analyser as previously described in [57] with some modifications. Briefly, ten age-synchronized day 5 and day 12 nematodes were collected into each well of the seahorse utility plate containing M9 buffer. Basal oxygen consumption rate, maximal and spare respiratory capacity of the nematodes were determined by normalizing to the number of worms per well.

### 2.9 *In vitro* non-enzymatic acetylation assay

Mitochondria extracted from mouse liver were incubated for 6 hours at 37°C in buffer containing 150mM NaCl and 50mM Tris-HCl (pH 8.0), supplemented with either 1.5mM acetyl-CoA or 1.5mM sodium acetate.

### 2.10 *In vitro* deacetylation assay

Mitochondrial extracts subjected to the *in vitro* non-enzymatic acetylation assay were incubated with recombinant GST-tagged SIRT3 in SDAC buffer (50mM Tris-HCl pH 9, 4mM MgCl_2_, 50mM NaCl, 0.5mM DTT) and 1mM NAD^+^ at 37°C for 3 hours with gentle shaking. The reaction was then stopped by the addition of 4x SDS loading buffer.

## Results

### Validation of acetylated lysine antibodies

**Fig 1:**
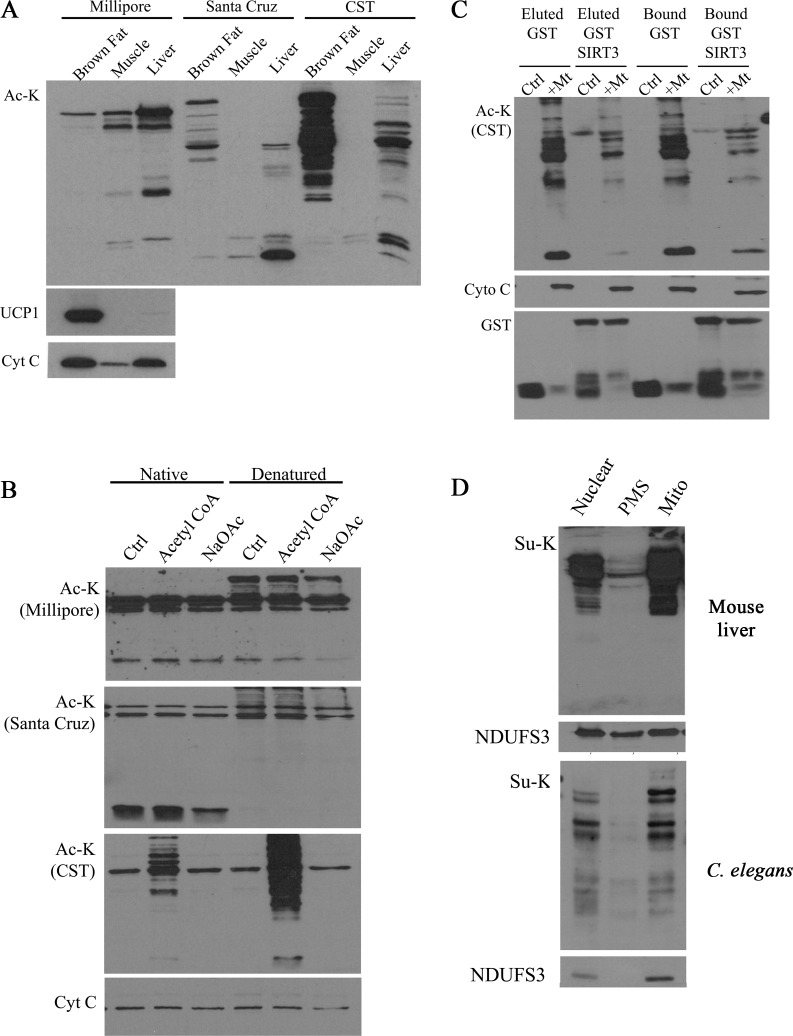
Validation of acetylated and succinylated lysine antibodies. (A-B) Three commercial anti-acetylated lysine antibodies i.e. 05–515 clone 4G12 (Millipore), sc-8649 (Santa Cruz), #9441 (CST) were compared by three different methods, i.e. (A) Mitochondrial lysates extracted from different mouse tissues were subjected to western blotting. (B) In vitro non-enzymatic acetylation assay using acetyl-CoA or sodium acetate (NaOAc; negative control) was conducted followed by western blot analysis. (C) In vitro deacetylation assay using mouse liver mitochondria and eluted recombinant GST-SIRT3 or GST-SIRT3 bound to GSH-agarose was performed and subsequently subjected to western blotting. (D) Subcellular fractionation of mouse liver homogenate or C. elegans was performed to isolate nuclear fraction (nuclear), postmitochondrial supernatant (PMS) and mitochondria enriched fraction (mito) for western blotting. Absence of signal in the PMS and a similar pattern in the nuclear and mito fraction that corresponds to the abundance of mitochondrial NDUFS3 indicates mitochondria-specific succinylation.

In our study, we were interested in studying two specific forms of acylation; acetylation and succinylation. As commercially available anti-acetylated lysine antibodies are raised against slightly different antigens, these antibodies likely have different specificity and sensitivity in detecting acetylated proteins. Because our studies were greatly dependent on high specificity and sensitivity, we initially compared anti-acetylated lysine antibodies from three different sources. We first tested the antibodies by blotting against mitochondrial extracts from mouse brown adipose, heart and liver tissues. We observed that antibodies from Santa Cruz and CST gave a similar band pattern, suggesting that these antibodies detected overlapping acetylated proteins (Fig 1A). In contrast, the Millipore antibody gave a very different band pattern. The Western blot results also suggest that the level of mitochondrial protein acetylation as well as the specific modified proteins vary between tissues.

We subsequently tested the antibodies in western blots of mitochondrial lysates subjected to an *in vitro* non-enzymatic acetylation reaction, as previously described by Wagner and Payne [47]. In this experiment, we incubated native or heat denatured mouse liver mitochondria in alkaline buffer supplemented with either 1.5mM acetyl-CoA or 1.5mM sodium acetate followed by western blot analysis. As shown in Fig 1B, a marked increase in acetylated proteins was detected in the Western blot probed with the CST antibody in mitochondria treated with acetyl-CoA, but not with the negative control sodium acetate. In contrast, no significant specific increase in the signal was observed with the Santa Cruz and Millipore antibodies, suggesting that these antibodies have lower sensitivity to detect protein acetylation. Of note, the results with the CST antibody also confirm previous findings by Wagner and Payne (2013) that non-enzymatic protein acetylation occurs upon incubation of mitochondria in the presence of acetyl-CoA in an alkaline environment. The increase in the level of acetylated proteins upon denaturing of proteins (which leads to the exposure of more lysine residues) is also consistent with a non-enzymatic acylation mechanism [47].

To further validate this approach, we then performed *in vitro* deacetylation assays to confirm that decreased mitochondrial protein acetylation levels in deacetylated samples could reliably be detected by our method. We initially subjected mitochondrial extracts to non-enzymatic acetylation and subsequently washed off the excess acetyl-CoA before incubating the mitochondrial lysates with either recombinant GST or recombinant GST-SIRT3 protein. As shown in Fig 1C, for mitochondrial lysate that was incubated with recombinant GST-SIRT3 protein, there was a marked uniform decrease in band intensity compared to lysate that was incubated with control recombinant GST protein. These results further validate the CST antibody, which was used in subsequent experiments to detect mitochondrial protein acetylation. Furthermore, the results also confirm that the signal detected after the *in vitro* incubation of mitochondria with acetyl-CoA at alkaline pH is indeed due to protein acetylation and that SIRT3 can efficiently remove this modification.

In order to measure protein succinylation we used the only commercially available antibody that has been used in previously published studies (Materials and Methods). When using subcellular fractions from mouse liver and *C*. *elegans*, we detected no significant protein succinylation of the postmitochondrial fraction, but abundant protein succinylation in the mitochondrial and somewhat less signal in the nuclear fraction. The band pattern in the nuclear fractions resembles that of the mitochondrial fraction, and the mitochondrial marker protein NDUFS3 was present in both the mitochondrial and nuclear fractions at a level that resembled that of the succinylated proteins. These results strongly suggest that the signal detected in the nuclear fraction is due to mitochondrial contamination. The preferential detection of succinylated proteins in mitochondria is consistent with previous findings [43] and also validates the antibody used in our study.

### Mitochondrial protein acylation increases with age in *C*. *elegans*

**Fig 2:**
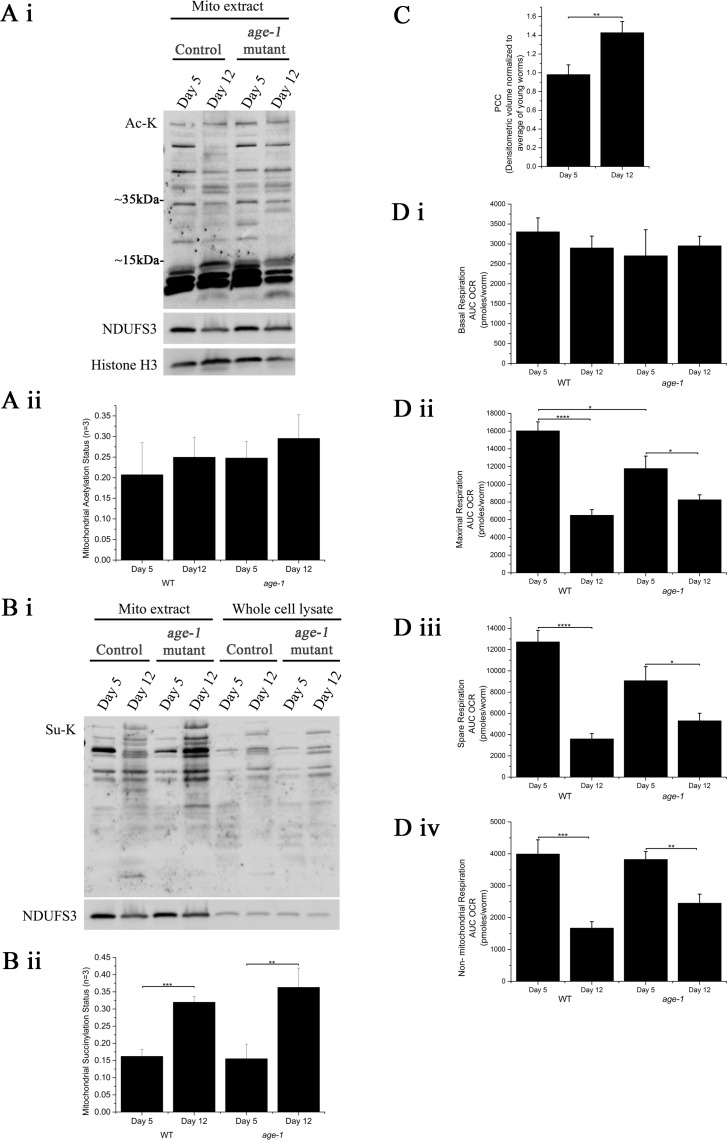
Mitochondrial acylation increases to a similar degree in carbonylation with ageing in *C*. *elegans*. Subcellular fractionations of *C*. *elegans* homogenates were performed followed by western blotting using anti-acetylated lysine antibody(A) or anti-succinylated lysine antibody(B). Protein carbonyl content was determined using slot blot followed by derivaziation and detection using anti-2,4-dinitrophenylhydrazine antibody(C). (Ai, Bi) The western blots shown are representative of three independent experiments. Densitometry was then performed to quantify the mitochondrial acylation status. The data shown in (Aii, Bii, Cii) represent the average (±SD) of three independent experiments. (D) Oxygen consumption of Day 4 and Day 12 worms measured using Seahorse flux analyzer. Bar graphs shown represent basal respiration (i), maximal respiration (ii), spare respiration (iii) and non-mitochondrial respiration (iv) of *C*. *elegans*. (A-D) Statistical analysis was performed using unpaired t-test, * = p≤0.05, ** = p≤0.01, *** = p≤0.001 and **** = p≤0.0001.

We first tested the hypothesis that acylation of mitochondrial proteins increases with age in N2 wild-type (WT) *C*. *elegans*. We extracted mitochondria from ageing WT animals followed by western blotting and quantification using densitometry and normalization against the mitochondrial loading control NDUFS3. When blotting for mitochondrial acetylation (Fig 2A i), we observed a very strong signal at low molecular weight (~20kda). It is likely that this signal represents acetylated histone proteins as we detected the presence of histone H3 contamination in the mitochondria enriched fraction. Hence, when quantifying the western blot results using densitometry, we excluded bands with a molecular weight of 20kDa or less. When analysing three independent experiments (Fig 2A ii), we observed an apparent (but non-significant) trend to accumulation of acetylated mitochondrial proteins between young (day 5) and old (day 12) WT *C*. *elegans*. To further explore the role of acylation in ageing we also determined age-dependent changes in mitochondrial protein acetylation in a long-lived mutant strain (*age-1*) of *C*. *elegans*. The *age-1* mutation was the first single gene mutation identified to significantly extend the lifespan of *C*. *elegans*. Mutant animals of this strain are long-lived, exhibit increased resistance to several stressors but also suffer from evolutionary trade-offs [58–60]. However, there was no difference in protein acetylation status between young and old WT or *age-1* animals (Fig 2A ii).

Given our observation that protein succinylation was significantly enriched in mitochondria, we expected that protein succinylation would occur preferentially in mitochondria of ageing *C*. *elegans* and therefore determined protein succinylation in whole worm lysate and mitochondrial protein extract of ageing WT and *age-1 C*. *elegans* (Fig 2B i). Protein succinylation was indeed significantly more prominent in mitochondrial protein than in total protein, similar to the mitochondrial marker protein NDUFS3. Furthermore, the band pattern of succinylated proteins was highly similar in the mitochondrial and total protein fraction, thus confirming preferential succinylation of proteins in mitochondria. For both WT and *age-1* animals protein succinylation showed a significant age-dependent increase in the mitochondrial extracts (Fig 2B ii) with a similar trends in whole worm lysate. Age-dependent increases in oxidative damage to protein are commonly measured using protein carbonyl content. Such protein modification by carbonylation is associated with ROS mediated damage and is well characterised to increase significantly with age in *C*. *elegans* [61,62]. To compare protein succinylation patterns with this, well-established type of modification, we therefore also determined protein carbonyl content in mitochondrial protein. As expected, protein carbonyl levels increased significantly with age in WT *C*. *elegans* (Fig 2C). Strikingly, the accumulation in protein succinylation was at least as large in magnitude compared to the accumulation in oxidatively modified protein as determined via protein carbonyl levels (Fig 2).

Given the hypothesis that non-enzymatic protein acylation may result in mitochondrial dysfunction, we then determined metabolic capacity in both old and young animals of both strains using a Seahorse metabolic analyser. This analyzer allows determination of basal oxygen consumption as well as maximal oxygen consumption and spare respiratory capacity, a measure of the extent to which mitochondria can upregulate flux through the ETC in response to increased metabolic need [57]. As expected, we found that, while basal respiration was comparable, maximal and spare respiratory capacity of old (day 12) worms were substantially lower compared to young (day 5) animals (Fig 2D ii and 2 iii). This drop between age 5 and 12 was more pronounced and statistically significant in WT but smaller in the slower ageing *age-1* animals. Interestingly, young *age-1* animals exhibit a significantly lower maximal respiratory capacity compared to WT, however, compared to WT, *age-1* animals experience a smaller age-dependent decline such that, by day 12, *age-1* animals show a trend towards having a higher maximal capacity than WT (Fig 2D ii). Taken together, our results are suggestive of age-dependent increases in mitochondrial protein acylation, in particular succinylation, in *C*. *elegans*. This age-dependent increase in succinylation appears more robust that than the well-established increase in protein carbonyl content in mitochondria. While *age-1* animals show similar acylation levels as WT animals, these data were consistent with the observation that young *age-1* animals exhibited lower spare respiratory capacity than young WT animals.

**Fig 3:**
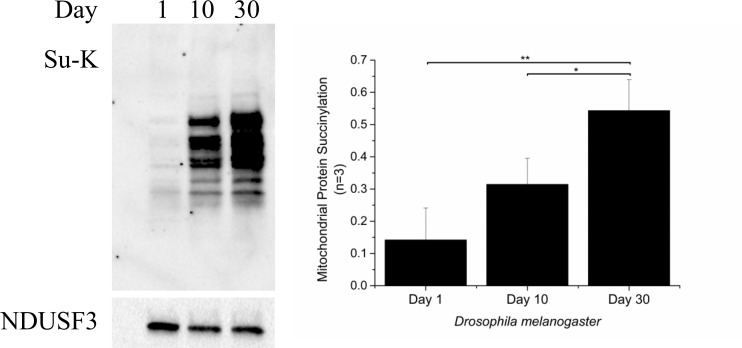
Mitochondrial succinylation increases with ageing in *D*. *melanogaster*. *D*. *melanogaster* were homogenized and the whole fly lysates were used for western blotting using anti- succinylated lysine antibody. The bar graph represents the average (±SD) of three independent experiments. Statistical analysis was performed using unpaired t-test, * = p≤0.05and ** = p≤0.01.

To further investigate age-dependent protein acylation to mitochondrial protein we used another short-lived, simple model organism, *D*. *melanogaster*. Similar to *C*. *elegans*, neither SIRT3 nor SIRT5 are present in this organism [63]. We collected flies on day 1, day 10 and day 30 and prepared total fly homogenates that were used for western blotting using succinyllysine specific antibody. We were unable to measure mitochondrial protein acetylation using the total fly homogenates because, in contrast to succinylation, protein acetylation is ubiquitous throughout the cell. As before, western blot results were quantified by densitometry and normalized to mitochondrial NDUFS3. Consistent with the results in *C*. *elegans*, we observed an apparent age-dependent increase in mitochondrial protein succinylation between day 1 to day 30 (Fig 3). This impression was confirmed statistically by analysing the densitometric data (P<0.01, One-way ANOVA). Together, these data show that there is a consistent pattern of increasing protein succinylation in mitochondrial proteins in both short-lived model organisms lacking mitochondrial sirtuins.

### Mitochondrial protein acylation does not increase with age in rats

**Fig 4:**
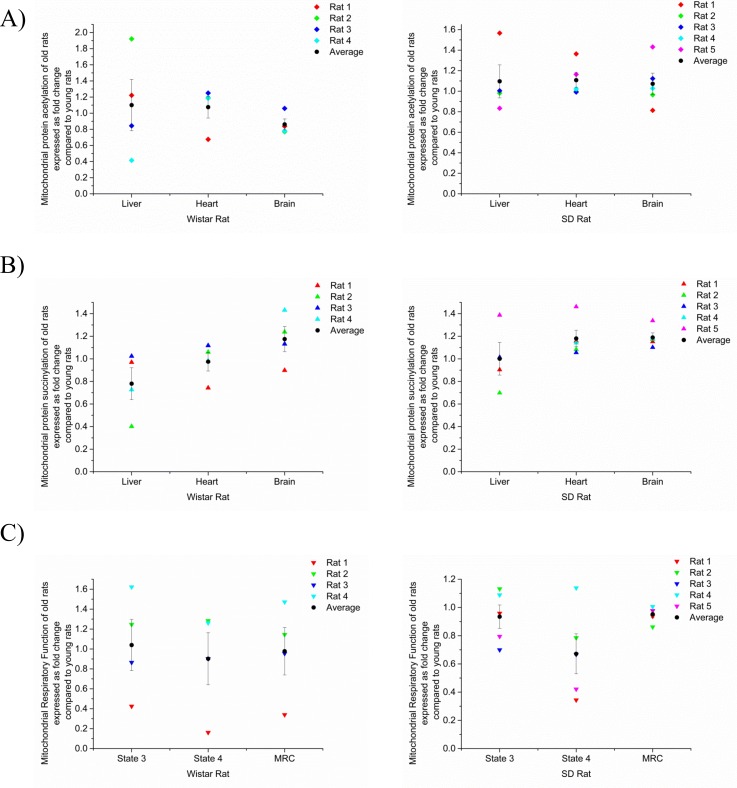
Mitochondrial acylation in young and old rats. Subcellular fractionations of rat liver, heart and brain tissues were performed followed by western blotting using anti- acetylated lysine (A) or anti-succinylated lysine (B) antibody. Densitometry was then performed to quantify the mitochondrial acylation status. (C) Subcellular fractionations of rat, i.e. Wistar and SD brain tissues were performed followed by oxygen consumption measurement using a Clarke electrode and the state 3, state 4 and maximal respiratory rates are shown. The data shown in (A, B, C) represent the average (±S.E.M), expressed as fold increase compared to young rat. Each data point represents an individual rat.

We then carried out analogous studies in rodents. For this part of the study, we used two different outbred rat strains, Wistar and Sprague Dawley (SD), for our experiments. We isolated liver, heart and brain tissues from 5 young Wistar and 4 young SD rats (1 or 1.5months old) and from 4 middle aged Wistar and 5 middle aged SD rats (18 or 11.5 months old) rats and subsequently extracted mitochondria. We carried out oxygen consumption measurements using mitochondria extracted from brain. We choose brain because of the significance of this tissue in ageing and age related diseases. However, despite the fact that neurons are post-mitotic cells and accumulation of mitochondrial damage, e.g. as a result of non-enzymatic protein acetylation, should be more readily observable, we detected no significant age-dependent differences in the state 3 respiration rate and the maximal respiratory capacity for either rat strains (Fig 4D), indicating that there was no significant mitochondrial decline between the ages of 1 and 18 month. We then determined protein acylation in isolated mitochondria of liver, heart and brain. As previously, we quantified the signal for each lane using densitometry and normalized against the mitochondrial loading control NDUFS3. The data are presented in the form of a dot plot together with the Western blot images (Fig 4). Each dot in the dot plot represents the acylation status of an individual rat. As shown in Fig 4, we observed a relatively wide distribution of the acylation status in all tissues of both the young and middle-aged groups, suggesting that mitochondrial protein acetylation varies between individual rats. However, in SD rats the data points were found to be more closely clustered (Fig 4A–4C). Nevertheless, overall, there were no age-dependent changes in mitochondrial protein acetylation and succinylation. These data are consistent with the lack of change in oxygen consumption rate over the same period of time.

## Discussion

The underlying hypothesis of our study is that an age dependent increase in mitochondrial protein acylation contributes to mitochondrial dysfunction during ageing and regulates organismal lifespan. This hypothesis is based on several well established observations in the literature: (1) Dysregulation of mitochondrial homeostasis has been implicated in ageing and a number of age-related diseases [12,64,65]. (2) A number of large scale proteomic studies have shown that mitochondrial proteins are highly acylated [29,43,66–68]. (3) In most cases, studies have shown that these modifications have an inhibitory effect on the function of the modified proteins and mitochondrial function overall [31,32,45]. Hence, our study sought to determine if there is any correlation between mitochondrial protein acylation and age using different model organisms.

Our results suggest that acylation, or more specifically succinylation, of mitochondrial proteins increases in an age dependent manner in *C*. *elegans* and *D*. *melanogaster* but not in rats. One explanation for this difference may be that, compared to *C*. *elegans* and *D*. *melanogaster*, mammals express several isoforms of the sirtuin family of NAD-dependent deacetylases, some of which are localized in mitochondria. Both rodents and humans express seven sirtuins, including SIRT1, the homolog of yeast Sir2, as well as three mitochondrial sirtuins, SIRT3, SIRT4 and SIRT5. In contrast, in *C*. *elegans* only SIRT1 and SIRT4 are present [63]. SIRT1 is mainly found in the nucleus. SIRT4, although localized to mitochondria, lacks significant deacetylase and desuccinylase activity [69] and reportedly functions as an ADP-ribosyl transferase or as lipoamidase regulating pyruvate dehydrogenase complex activity [63,70,71]. Whether it can also function as a deacetylase is currently controversial. On the other hand, mitochondrial SIRT3 and SIRT5, which catalyse the removal of acetyl- and succinyl-groups from lysine residues, respectively, are absent in *C*. *elegans* [63]. This may also explain why there is no difference between *age-1* and WT in terms of the rate of protein succinylation. While the long-lived *age-1* phenotype is associated with increased stress resistance and reduced damage accumulation, *C*. *elegans* may just lack any mechanism to remove succinylation. The increase in mitochondrial protein succinylation was comparable in magnitude to the accumulation of oxidative damage as determined as protein carbonyl content (Fig 2). Both oxidative damage and protein succinylation are expected to negatively affect protein function and the significant increase in succinylation therefore suggests that protein succinylation might affect mitochondrial function to a similar extent as oxidative damage.

We observed that the number of strongly acylated mitochondrial proteins is relatively low. This is in contrast to the findings reported by large-scale mass spectrometry studies that have identified large numbers of modified proteins [29,43]. However, this is most likely due to methodological differences because, unlike mass spectrometry, western blotting predominantly detects proteins that are acylated at relatively high stoichiometry. In contrast, recent mass spectrometry based studies in *Saccharomyces cerevisiae* reported that the vast majority of mitochondrial and cytoplasmic acetylation and succinylation occurs at very low stoichiometry [30,48,50]. It might therefore not be surprising that the number of strongly acylated mitochondrial proteins detected by Western blot is relatively low. Such highly modified proteins are, however, likely to be associated with be the most physiologically significant changes during ageing and it might be useful to determine age-dependent changes in stoichiometry of mitochondrial protein acylation.

We subsequently attempted to test whether mitochondrial protein acylation increases with age in mammals by using two different strains (Wistar and SD) of rat. For the Wistar strain, we used 1 month old versus 18 months old rats while for the SD strain we compared 1.5 months old versus 11.5 months old rats. However, we did not observe significant changes in the acylation status in most of the tissues of either rat strain. We were unable to detect any significant decline in mitochondrial function in these animals. A potential explanation for this observation is that there was simply not enough time for animals to develop significant age-dependent mitochondrial decline and it is therefore possible that both our failure to detect clear differences in acylation and oxidative phosphorylation activity was due to the suboptimal age ranges of the cohorts used in our studies. However, rats also possess seven sirtuins (SIRT1-SIRT7), including mitochondrial SIRT3 and SIRT5. In contrast, nematodes and many arthropods (e.g. *D*. *melanogaster*) lack mitochondrial sirtuins and thus are deficient in mitochondrial deacetylase and desuccinylase activities [63]. Another explanation for the lack of increase in these modifications, even over the course of 10 months, may therefore be that mitochondrial sirtuins efficiently prevent accumulation of acylated mitochondrial proteins during ageing. Consistent with this, it has recently been shown that Sirt3 in mice is essential to maintain mitochondrial protein acetylation at very low stoichiometry [48,50,72]. Finally, in addition to differences in sirtuin deacetylase expression it is also possible that the differences between nematodes, fruit flies and rats are due to intrinsic differences in mitochondrial acetyl-CoA and succinyl-CoA concentrations. It is well known that the mitochondrial acetyl-CoA and succinyl-CoA concentrations undergo changes under physiological and pathological conditions that are accompanied by changes in mitochondrial protein acylation. For instance, fasting, resulting in increased acetyl-CoA generation from free fatty acids in the liver, results in increased liver mitochondrial protein acetylation [49,50]. Mutations in NADP^+^ isocitrate dehydrogenase in cancer, which are known to lead to the accumulation of the oncometabolite R-2-hydroxyglutarate, result in competitive inhibition of succinate dehydrogenase, accumulation of mitochondrial succinyl-CoA and hypersuccinylation of mitochondrial proteins [73]. It is thus possible that age dependent increases in mitochondrial protein acylation are dependent on changes in mitochondrial acetyl-CoA and succinyl-CoA concentrations. Of note, a recent study has found that early ageing is associated with elevated acetyl-CoA concentrations in flies [42]. This also raises the possibility that altered expression or activities of human SIRT3 and SIRT5 may contribute to ageing in humans, especially as human SIRT3 and SIRT5 genes have been associated with survival to old age and ageing brain health [74–76].

## Supporting Information

S1 Fig Mitochondrial acylation in young and old rats. Subcellular fractionations of Wistar (A) and SD (B) rat liver, heart and brain tissues were performed followed by western blotting using anti- acetylated lysine or anti-succinylated lysine antibody.
(TIF)
