# Supplementary figures and images for: The Role of Mitochondrial Non-Enzymatic Protein Acylation in Ageing
Source: PLoS One. 2016 Dec 29;11(12):e0168752. doi: 10.1371/journal.pone.0168752 (PMC5199114; doi:10.1371/journal.pone.0168752)

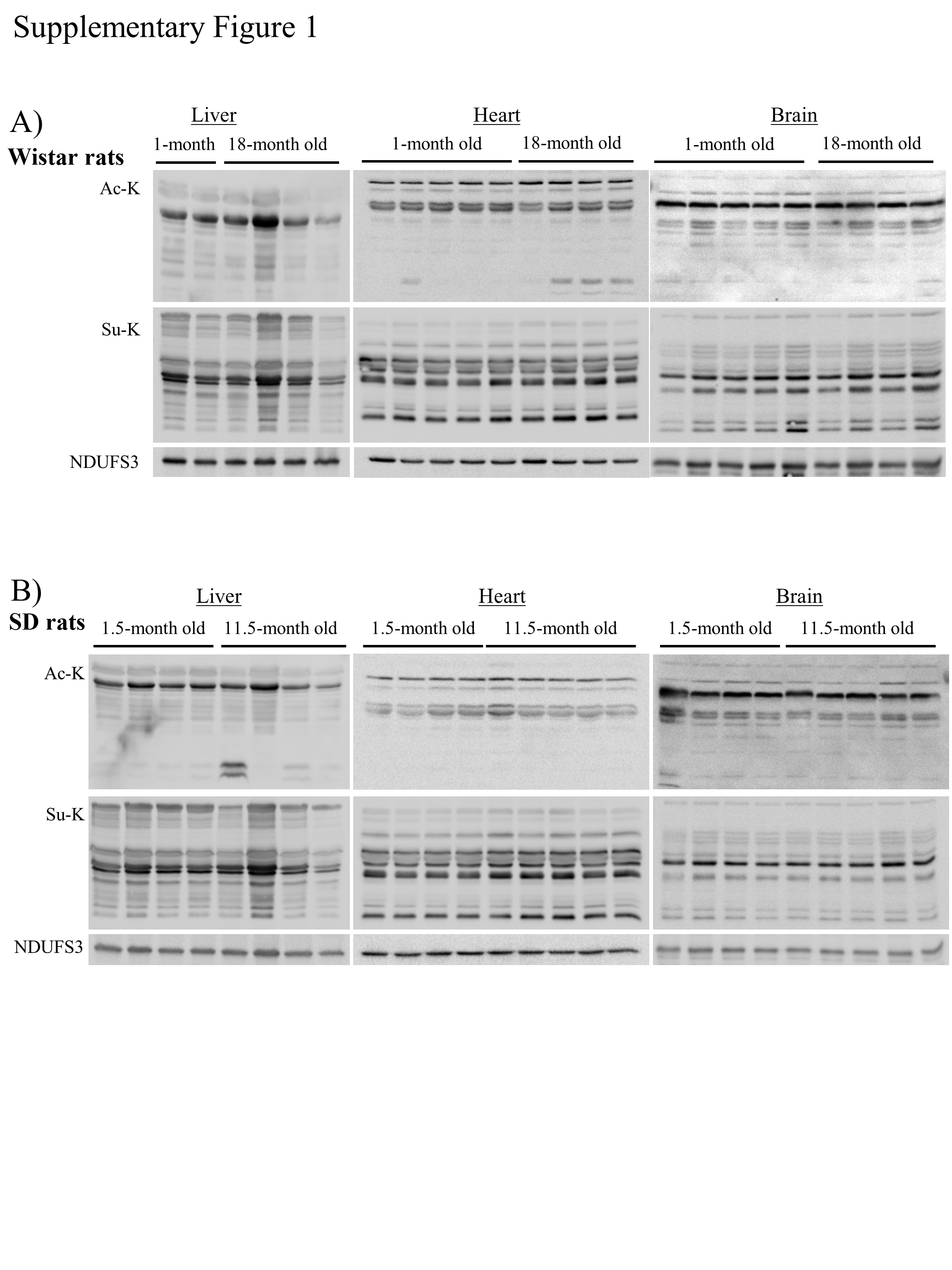

Supplement: S1 Fig — Subcellular fractionations of Wistar (A) and SD (B) rat liver, heart and brain tissues were performed followed by western blotting using anti- acetylated lysine or anti-succinylated lysine antibody. (TIF) [file pone.0168752.s001.tif]
